# Role of Flexible Bronchoscopy using Biopsy Forceps as the Initial Attempt for Headscarf Pin Aspiration Extraction

**DOI:** 10.2174/18743064-v17-e230718-2023-5

**Published:** 2023-08-07

**Authors:** Mia Elhidsi, Dicky Soehardiman, Wahju Aniwidyaningsih, Mochamad Fahmi Alatas, Ginanjar Arum Desianti, Prasenohadi Pradono

**Affiliations:** 1 Department of Pulmonology and Respiratory Medicine, Universitas Indonesia, Persahabatan Hospital, Jakarta, Indonesia

**Keywords:** Aspiration, Biopsy forceps, Bronchoscopy, Flexible bronchoscopy, Foreign bodies, Headscarf pin

## Abstract

**Introduction:**

Flexible bronchoscopy is a less invasive procedure for extracting foreign bodies from the airways. However, studies on the extraction of headscarf pins are still very limited to determine the efficacy and safety of headscarf pin extraction using flexible bronchoscopy with biopsy forceps.

**Methods:**

This retrospective study was conducted at Persahabatan Hospital, Jakarta, Indonesia, on patients who had been treated in this hospital for headscarf pin extraction between January 2013 and February 2023. Fibreoptic bronchoscopy was performed under general anaesthesia. The pin was removed using Radial Jaw 4 mm single-use pulmonary biopsy forceps. The impacted sharp tip of the pin was freed first, and the proximal part of the pin body was gripped using biopsy forceps. Once a firm hold of the sharp end or the proximal part of the pin was secured, the bronchoscope and forceps were both slowly withdrawn under direct vision.

**Results:**

Thirty-two cases with headscarf pin aspiration were managed by fibreoptic bronchoscopy. A total of 12 patients (37.5%) came without any respiratory complaints; however, an equal number complained of cough and 6 cases (18.7%) of haemoptysis. All the cases in which the pins were visible in the airway were found with the round head down and the sharp tip oriented superiorly in the airway and impacted in the mucosa. Fibreoptic bronchoscopy extraction succeeded in 31 cases (96.8%). Only one case was converted to surgery. There were no major complications.

**Conclusion:**

Fibreoptic bronchoscopy with biopsy forceps under general anaesthesia is safe and effective for the removal of headscarf pin aspiration.

## 1 INTRODUCTION

**Fig. (1):**
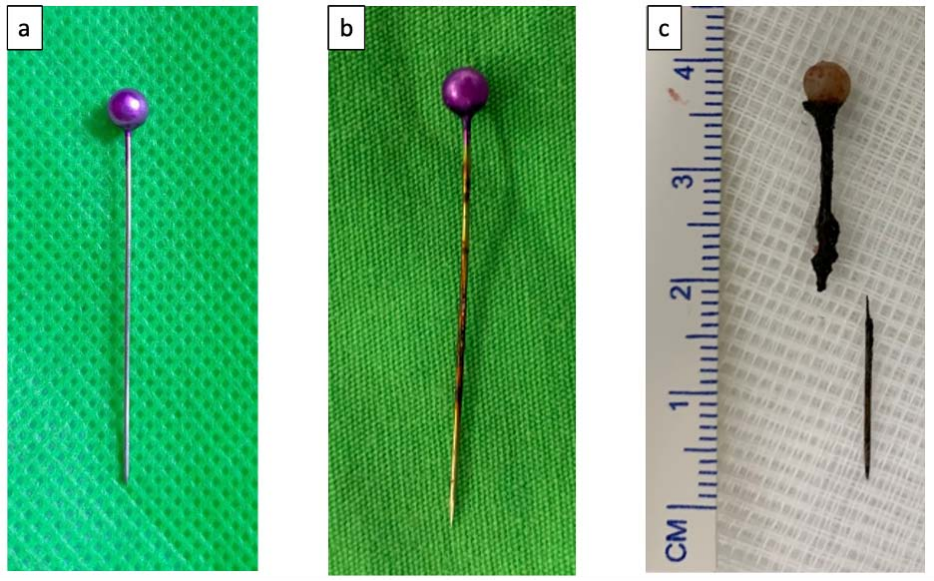
Aspirated headscarf pin. (**a**). Normal headscarf pin. (**b**). Corrosive headscarf pin. (**c**). Corrosive and broken headscarf pin.

Foreign body aspiration is a serious health problem that often occurs; however, it is preventable [1]. Foreign body aspiration is the event of inhaling an object into the airway and has the potential to be life-threatening [2]. In general, the incidence of foreign body aspiration is influenced by many factors including age, gender, occupation, geographical conditions, social and cultural factors, economic status, and eating habits [2]. Certain cultural aspects such as the use of pins by Muslim women to fasten a headscarf (a type of head covering) are risk factors for aspiration for such groups [3]. The pins are about 3 to 5.5 cm (average 4 cm) long and have a sharp tip at one end and a round head with a variety of pearl-like colours at the other end (Fig. **1**). The risk occurs because these women often put pins in their mouths while both their hands are occupied with tidying up the headscarves before pinning them down and may accidentally aspirate them [1, 4].

Flexible bronchoscopy is a relatively easy, less invasive, and safe procedure for extracting foreign bodies from the airways compared to rigid bronchoscopy and surgery; it also has a fairly high success rate [5, 6]. However, studies on the extraction of headscarf pins are still very limited. The appearance of the aspirated headscarf pin has certain clinical characteristics that require special techniques in the extraction process *via* flexible bronchoscopy. We conducted this study to determine the efficacy and complications of headscarf pin extraction using flexible bronchoscopy with biopsy forceps. We also present some of the techniques for using biopsy forceps in the extraction of headscarf pins.

## 2 MATERIALS AND METHODS

This retrospective study was conducted at the Indonesian National Referral Hospital for Respiratory Diseases, Persahabatan Hospital, on patients who had been treated in this hospital for headscarf pin extraction between January 2013 and February 2023. Diagnosis of headscarf pin aspiration was confirmed by typical history and chest X-ray or computed tomography (CT) within 24 hours before admission to the hospital emergency room. We used secondary data from hospital medical records and bronchoscopy reports.

### 2.1 Headscarf Pin Removal

After conducting a history and physical examination of each patient with a history of headscarf pin aspiration, radiological examinations were conducted using chest X-rays or CT. The aspirated headscarf pin appears as a radiopaque, needle-like object in the airway.

Fibreoptic bronchoscopy was performed under general anaesthesia on the day of the admission of the patients. The bronchoscope was passed through the laryngeal mask airway (LMA), and the pin was removed using Radial Jaw 4 mm single-use pulmonary biopsy forceps. The sharp tip of the pin could be impacted in the mucosa and had to be freed first. If the sharp tip was free or had been released, then the sharp end of the proximal part of the pin body was gripped using biopsy forceps. Once a firm hold of the sharp end of the proximal part of the pin was secured, the bronchoscope and forceps were both slowly withdrawn under direct vision. After the pin passed through the vocal cords and reached the LMA, the LMA, and scope were lifted together. It is also possible to lift the pin completely until it leaves the LMA. After the removal of the pin, the bronchoscope was passed again to evaluate any possible post-extraction damage to the vocal cords and tracheobronchial mucosa. The process of headscarf pin extraction using biopsy forceps is shown in Fig. (**2**).

### 2.2 Data Extraction and Analysis

We used secondary data from hospital medical records, bronchoscopy reports, and videos. Removal time starts from the appearance of the biopsy forceps on the monitor until the pin is in the LMA. Descriptive data are presented in the form of amounts, percentages, and median values. We have also presented the drawings of removal techniques. An ethical review was not required for this study.

## 3 RESULTS

Thirty-two cases with headscarf pin aspiration were managed by flexible bronchoscopy as an initial attempt. The ages of patients ranged from 2 to 53 years, with a median age of 13 years. Additionally, although headscarf pins are generally used by women, there were 6 male patients (18.7%). In most of the cases – 26 cases were (81.2%) –the patients arrived at our centre more than 24 hours from the time of the aspiration event. All the patients were in good clinical condition and without any respiratory distress. A total of 12 patients (37.5%) came without any respiratory complaints; however, an equal number complained of cough and 6 cases (18.7%) of haemoptysis. All the cases in which the pins were visible in the airway were found with the round head down and the sharp tip oriented superiorly in the airway and impacted in the mucosa. In two of the cases, the pin was not seen during the bronchoscopy evaluation. The bronchoscopy view of headscarf aspiration is presented in Fig. (**3**). Some of the headscarf pins fell into the right basal lobe and were lodged there; this was followed by the right main bronchus, left main bronchus, right lower lobe, and trachea. None of the pins fell into the left upper lobe. The proportions of aspirated pins at various locations are shown in Fig. (**4**). Eight cases (25%) showed mucosal granulation, while one case had mucoid mucus at the proximal of the lumen.

Flexible bronchoscopy extraction succeeded in 31 cases (96.8%). Of the 17 cases whose extraction procedure videos were recorded, the average procedure duration was 5 minutes and 6 seconds. Only one case was converted to surgery; however, there were no major complications. In this case, the headscarf pin had been aspirated three years previously. The pin had fallen peripherally in the right lower lobe with the oedematous lumen and could not be visualized on the bronchoscopy image. Bronchoscopy was performed under fluoroscopy guidance. Some of the brittle pin parts were successfully removed using biopsy forceps, while the rest of the pin was removed by slashing the lung parenchyma and bronchi until the pin could be removed. After the procedure, the patient’s clinical condition was good and haemodynamically stable. One other case with no visual pin on the bronchoscopy monitor also required c-arm fluoroscopy. Chest X-ray and post-bronchoscopy administration of antibiotics is not a standard procedure in our center and is performed only with the indication. The observation period was carried out for 24 hours. Furthermore, the patient can be discharged if stable.

## 4 DISCUSSION

Headscarf pin aspiration has unique clinical characteristics. Considering that pins are mostly used by young adult women and those in their teens, the proportion is higher among women. In pin aspiration, there is always a history of aspiration. Unlike other foreign bodies, aspiration of a headscarf pin does not cause central airway obstruction but can trigger local mucosal reactions, hypersecretion, infection, inflammation, and the formation of granulation tissue around the needle as a result of foreign bodies penetrating the mucosa [7, 8]. Pin aspiration can produce a penetrating syndrome, which consists of three clinical phases: choking, followed by sudden episodes of coughing and dyspnoea, and finally ending in an asymptomatic or quiescent phase [4, 9-13]. However, pin aspiration is also dangerous because it can move suddenly and can penetrate the bronchial wall to the lung parenchyma [13]. The longer a needle is in the airway, the higher the possibility of the needle moving to the distal part so it becomes more difficult to remove it [14].

The history of headscarf pin aspiration is similar in most cases, and, before the incident, most of the subjects had inserted the head of the needle inside the mouth, and the sharp tip was outside. Therefore, most needles that enter the airway will generally have a similar orientation, that is, the sharp tip will be pointing up. Then, after the needle has been aspirated and the patient’s cough reflex appears, the needle is pushed upwards and the sharp tip will stick in the airway mucosa [3]. There is no specific predilection site, and this study found that the most common location was the right lower lobe bronchus. This is because of the anatomical structure of the right main bronchus, which is wider and more vertical than the left main bronchus [15, 16]. However, the left main bronchus, which has a smaller diameter, can generate greater negative pressure so that the needle will enter the left main bronchus more often.

Our study results show a high success rate with no major complication of flexible bronchoscopy using biopsy forceps as the initial attempt for headscarf pin aspiration extraction. Bronchoscopy is the main treatment modality for the extraction of aspirated pins but requires a skilled operator because the needle moves easily. Successful management of bronchoscopy reduces the need for surgical procedures such as thoracotomy, bronchotomy, or lobectomy. The use of Laryngeal mask airways (LMA) is preferred over endotracheal tube (ETT) because there is a risk that the pin will get stuck at the end tip of the ETT, and make the extraction more difficult.

Most cases can be treated with bronchoscopy. Only 0.9–13.9% of cases require surgical procedures [4, 9, 11, 14, 17, 18]. The determining factors for successful extraction using bronchoscopy include experienced operators, a more proximal anatomical location, and a fast duration of intervention. Nonetheless, surgical procedures are more often performed on needle aspiration patients than on other foreign body aspiration patients because a needle is smaller and thus easier to move distally. Moreover, if there are complications such as the growth of granulation tissue and mucus plugging, it becomes difficult to remove the pins using bronchoscopy.

Some articles report that the use of rigid bronchoscopy is more desirable than flexible bronchoscopy [1, 7-11, 19]. Rigid bronchoscopy is the main choice for sharp foreign body extraction as it has a low risk of perforation and bronchial injury, can maintain airway patency properly, and facilitate the extraction process because it can be done simultaneously making the procedure quicker [1, 13]. For adult patients, flexible bronchoscopy can be considered the first-line treatment, while still providing rigid bronchoscopy and further intervention facilities if extraction using a flexible bronchoscope fails [20]. Flexible bronchoscopy is useful because of its simple, smooth, and slender shape and its ability to pick up a needle that is more distally located, reducing the length of stay in the hospital. When it is not possible to retrieve the needle *via* flexible bronchoscopy, the needle can be localized, as was the case in one of the patients in this study wherein bronchotomy was resorted to [1, 3, 10].

**Fig. (3):**
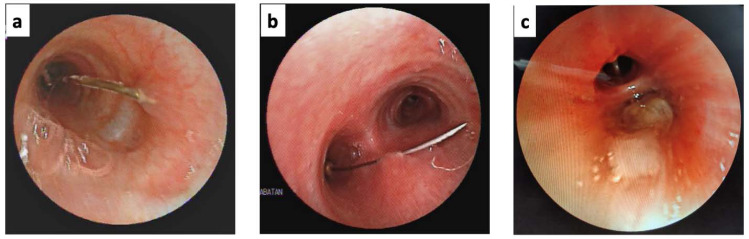
Bronchoscopy view of headscarf aspiration. (**a**). Headscarf pin was at left main bronchi with impacted sharp tip. (**b**). Headscarf pin crossed the proximal trachea with the pin body embedded in the posterior tracheal wall. (**c**). Headscarf pin was not visible, covered with mucoid secretion.

The main principles in removing a needle with the help of a bronchoscope are to hold the sharp end so that when the needle is removed it does not injure the mucosa and to concentrate the visualization of the needle in the middle of the airway (Fig. **3**). Previous studies have reported that needles can be removed using forceps or magnetic extractors [12]. Several types of forceps are used, including biopsy forceps, rat tooth forceps, or disposable grasping forceps [8]. In general, only one bronchoscopy procedure is needed to remove the needle. Further trials are required in 4.1–6.8% of cases [4, 17]. Fluoroscopy or X-ray equipment should be available as it will be necessary to localize the needle in case the needle moves, is accidentally removed during the procedure, and cannot be seen in the airway on standard bronchoscopy, which happened in one of the cases in this study.

Pin aspiration can be prevented by not putting needles in one’s mouth. The pin should be placed in special pads or magnetic containers [4]. Another alternative to prevent aspiration is to secure the headscarf using a device other than a needle, such as by tying it or using adhesives, clamps, or safety pins [9, 11]. Nevertheless, if someone has an aspiration, they should be taken immediately to the hospital so that it can be treated immediately [10, 21]. All this information needs to be conveyed to all young girls in their schools or through the public media to educate them and increase their awareness regarding the problem of pin aspiration.

## CONCLUSION

Headscarf pin aspiration has unique clinical characteristics. Most of the time, the needles orient themselves in a position where the round head is down and the sharp part is stuck in the mucosa. Needle extraction using biopsy forceps is preceded by releasing the mucosa where the needle is stuck and then grasping it and finally pulling the forceps together with a flexible scope by keeping the scope in the middle and preventing the sharp tip from injuring the airway. Fibreoptic bronchoscopy with biopsy forceps is safe and effective for the removal of headscarf pin aspiration.

## AUTHORS’ CONTRIBUTIONS

ME, DS, WA, MFA, GAD, and PHD managed the patients. ME drafted and submitted the manuscript. ME, DS and MFA revised the manuscript. DS and PHD supervised the work.

## Figures and Tables

**Fig. (2):**
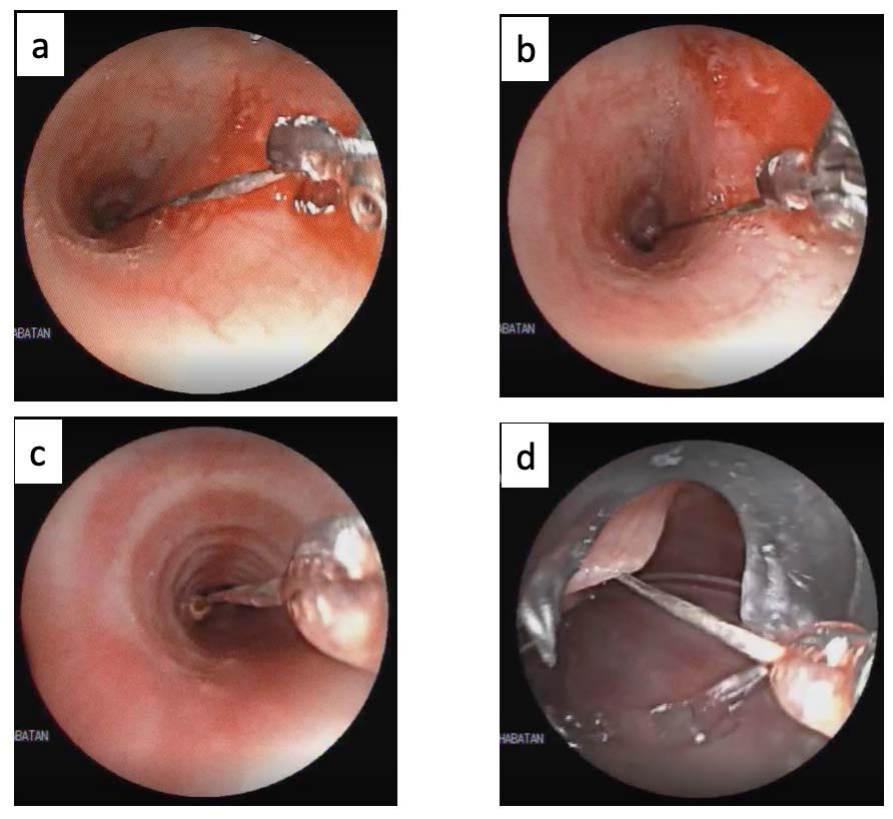
Headscarf pin extraction using biopsy forceps. (**a**). Release the impacted sharp tip from the mucosa. (**b**). Grip the proximal part of the pin with biopsy forceps. (**c**). Once a firm hold of the proximal pin is secured, withdraw the bronchoscope and forceps slowly under direct vision. (**d**). After the pin passes through the vocal cords and reaches the laryngeal mask airway (LMA), lift the LMA and scope together. LMA: Laryngeal mask airway.

**Fig. (4):**
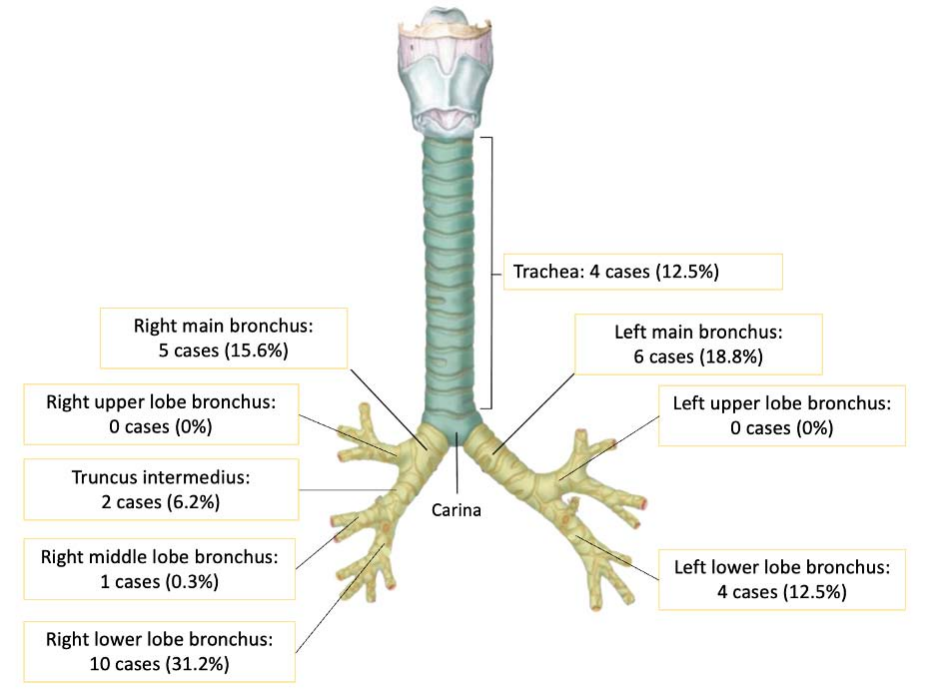
Distribution of headscarf pin aspiration in the airway.

## Data Availability

The data that support the findings of this study are available from the corresponding author, [ME], on correspondence request.

## LIST OF ABBREVIATIONS

CT: = Computed Tomography
LMA: = Laryngeal Mask Airway
